# Encircling Scleral Buckling Surgery for Severe Hypotony with Ciliary Body Detachment on Anterior Segment Swept-Source Optical Coherence Tomography: A Case Series

**DOI:** 10.3390/jcm11164647

**Published:** 2022-08-09

**Authors:** Sławomir Cisiecki, Karolina Bonińska, Maciej Bednarski

**Affiliations:** 1 Centrum Medyczne “Julianów”, ul. Żeglarska 4, 91-321 Lodz, Poland; 2 Miejskie Centrum Medyczne, ul. Milionowa 14, 93-113 Lodz, Poland

**Keywords:** ciliary body, hypotonia, intraocular pressure, optical coherence tomography, proliferative vitreoretinopathy, retinal detachment, scleral buckling

## Abstract

This study aimed to evaluate the usefulness of an encircling scleral buckling procedure to manage severe hypotony secondary to proliferative vitreoretinopathy (PVR)-induced retinal detachment. This retrospective study included six eyes of six patients (five women and one man) with hypotony (intraocular pressure [IOP] ≤ 6 mmHg) after multiple reattachment surgeries for PVR-induced retinal detachment. In patients with failure of hypotony resolution after conservative treatment (dexamethasone drops five times daily), 360° scleral buckling was performed under periocular anesthesia. The light perception was evaluated immediately postoperatively. The anatomic parameters were evaluated pre- and postoperatively observed on anterior segment swept-source optical coherence tomography. Ciliary body detachment (CBD) secondary to advanced cyclitic membranes associated with PVR grades C and D was detected in all eyes with hypotony. The mean IOP increased in all eyes (4.83 mmHg preoperatively vs. 10.17 mmHg postoperatively, *p* = 0.006), with subsequent improvement in best-corrected visual acuity (1.91 logMAR preoperatively vs. 1.50 logMAR postoperatively, *p* = 0.034). However, no eye showed any significant changes in CBD postoperatively. Scleral buckling surgery might be useful to increase IOP in eyes with persistent severe hypotonia secondary to PVR-induced CBD. Further studies are needed to improve outcomes in eyes with severe PVR-induced retinal detachment.

## 1. Introduction

Currently, ultrasound-guided biomicroscopy (UBM) and anterior segment optical coherence tomography (OCT) are the main tools available to visualize ciliary body structures. Ciliary body clefts can also be visualized using gonioscopy. Each of these methods has its limitations [1]. Recently, high-resolution anterior segment swept-source OCT (AS-SS-OCT), in which an increased laser wavelength allows the imaging of previously invisible structures, has become available for accurate diagnostic imaging. The opacity of optical media and short examination time are the advantages of this technique [2].

The condition of the ciliary body in patients with persistently low intraocular pressure (IOP) was assessed using AS-SS-OCT. This condition can lead to hypotonic maculopathy, choroidal detachment, and irreversible visual loss. The treatment options described in the literature include ciliary body suturing, external transconjunctival cryotherapy, direct cyclopexy, ciliochoroidal diathermy, scleral buckling, vitrectomy with gas, and injection of a high-molecular-weight viscoelastic substance into the anterior chamber. Such treatments have mostly been applied to traumatic cases with cyclodialysis cleft [3,4,5,6,7].

We describe outcomes of scleral buckling surgery, which may prevent and treat persistent hypotony secondary to PVR-induced ciliary body detachment by anatomical closure of the cleft and restoration of the apposition of the ciliary body to the sclera.

## 2. Materials and Methods

**Figure 1:**
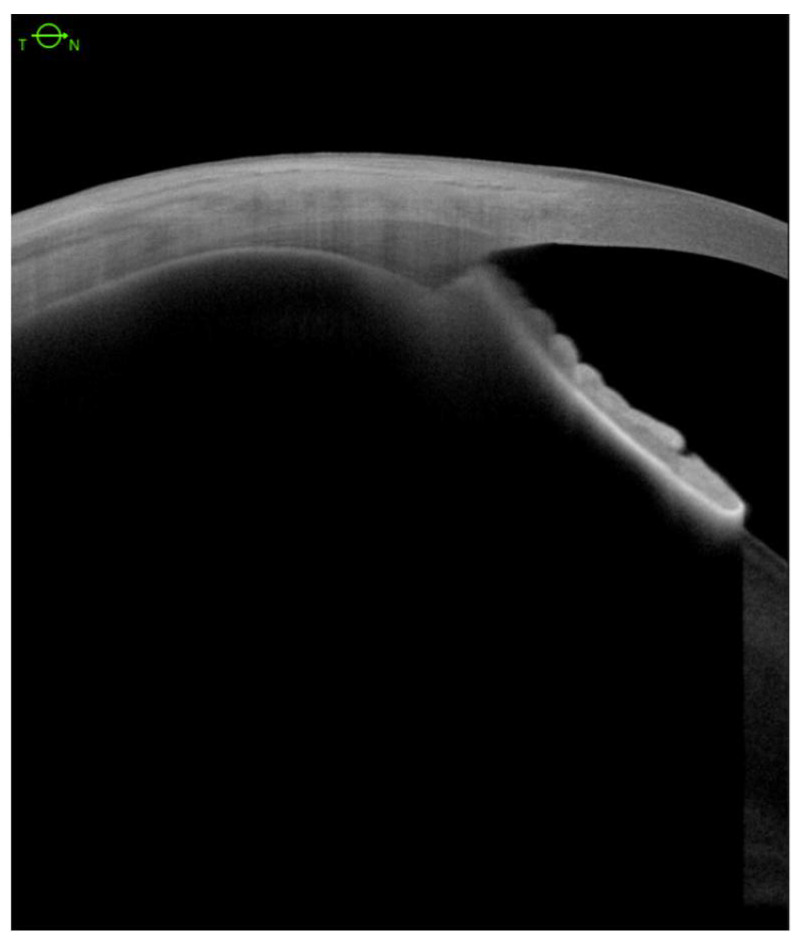
Anterior segment swept-source OCT with normal ciliary body structure.

Ciliary body structures in patients were analyzed with severe hypotony following multiple surgeries to correct retinal detachment. Anatomical outcomes were assessed using AS-SS-OCT images (Figure 1).

This retrospective, single-center case series study was conducted at the Ophthalmology Department at Dr Jonscher Hospital in Lodz. The study was in accordance with the declarations of Helsinki and the institutional guidelines.

Data from six eyes of six patients (five women and one man) with hypotony (IOP ≤ 6 mmHg) after multiple reattachment surgeries for proliferative vitreoretinopathy (PVR)-induced retinal detachment were retrospectively analyzed. The mean age was 65.6 years (range 48–82 years). Complete ophthalmic examination was performed preoperatively at 1 week and at 1, 3, 6, 9, and 12 months after surgery. Detailed patient characteristics are shown in Table 1.

Numerical traits were depicted by their arithmetical mean, median, and standard deviation values. Normality of distribution was assessed by using the Shapiro–Wilk test. Considering the small size of the study group, the Quade test with bootstrapping was used to assess the significance of differences in preoperative versus postoperative logMAR and IOP. A level of *p* < 0.05 was deemed statistically significant. All the statistical procedures were performed using IBM^®^ SPSS^®^ Statistics, version 28 (Armonk, New York, NY, USA).

All patients underwent a complete ophthalmic examination. Visual acuity was assessed using the ETDRS charts, and IOP was measured using Goldmann applanation tonometry. The ciliary body was examined using SS-OCT ANTERION^®^ (Heidelberg Engineering, Heidelberg, Germany).

As the hypotony did not resolve after conservative treatment (dexamethasone drops five times daily), surgical intervention with periocular anesthesia was performed. All patients provided written informed consent prior to surgery.

### 360° Scleral Buckling Surgery

The eye was encircled with a #41 silicone band after placing mattress sutures using 6-0 polyester in all four quadrants. Broad sutures were placed over the portion of the buckle to provide optimal pressure. This was a nondrainage procedure. The silicone band was tied above the most detached ciliary body quadrant at the equator level. At the end of the procedure, the light perception was evaluated.

## 3. Results

**Figure 2:**
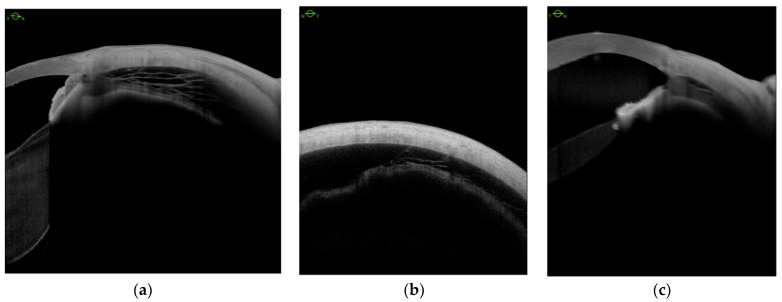
Anterior segment swept-source OCT. Ciliary body detachment between three types of fibers (**a**); supraciliary fluid in four quadrants in varying volume (**b**) and in the *pars plicata* (**c**).

**Figure 3:**
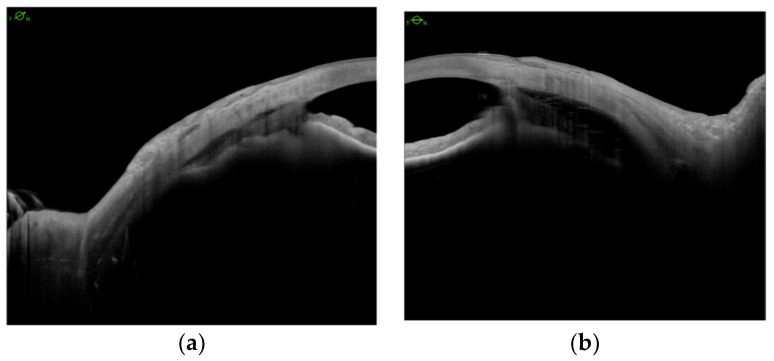

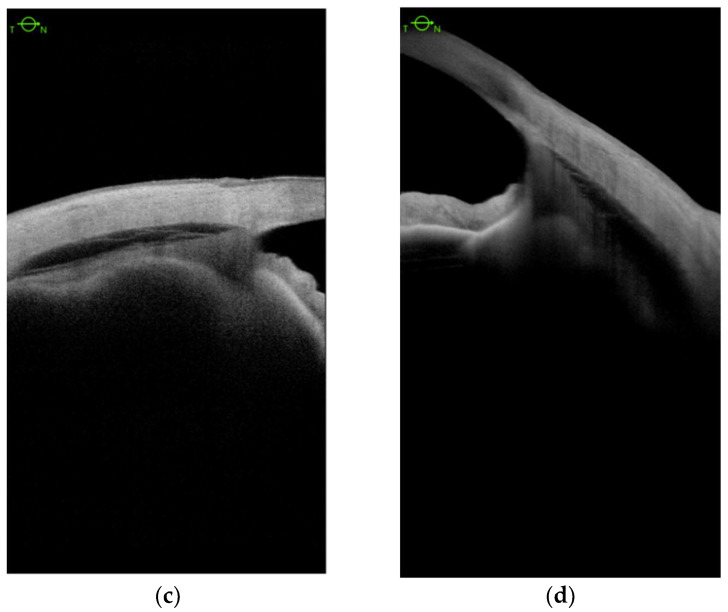
Anterior segment swept-source OCT demonstrates ciliary body detachment preoperatively (**a**,**b**) and postoperatively (**c**,**d**).

Ciliary body detachment (CBD) secondary to the advanced cyclitic membrane associated with PVR grades C and D was detected in all eyes with hypotony (Table 1). We defined three patterns of CBD: (1) detachment between the longitudinal, circular, and oblique fibers (Figure 2a); (2) complete detachment with supraciliary fluid of varying thicknesses in all 4 quadrants (Figure 2b); (3) detachment in the *pars plicata* (Figure 2c).

We analyzed CBD before and after scleral buckling surgery (Figure 3a,b), and no eye showed any significant changes postoperatively (Figure 3c,d).

Next, we analyzed the effect of surgery on IOP and BCVA. The mean IOP increased in all eyes (4.83 mmHg preoperatively, Me = 4.50; SD = 0.98 vs. 10.17 mmHg postoperatively, M = 10.17, Me = 9.50; SD = 3.37; *p* = 0.006), with subsequent improvement in best-corrected visual acuity (1.91 logMAR preoperatively, Me = 2.28, SD = 0.59, vs. 1.50 postoperatively, Me = 1.41, SD = 0.59; *p* = 0.034) (Table 1). 

## 4. Discussion

Using AS-SS-OCT, we assessed the condition of the ciliary body and its correlation with decreased IOP. UBM, the alternative method, may not have allowed us to obtain the high-resolution images and quantitative data obtained in this study using AS-SS-OCT. UBM requires immersion of the eye and a qualified technician to obtain high-quality images. Although newer UBM devices do not require immersion, direct contact with the eye is still required and can cause artifacts [8].

Visualized CBD creates abnormal pathway for aequos homor drainage into the suprachoroidal space and leads to hypotony [9]. Surgical management in such cases is indicated when medical treatment of hypotony fails. There are a few different techniques described to treat this complication [3,4,5,6,7] but there is no effective method in the literature to treat hypotony after PVR-related CBD.

In our study, IOP increased after scleral buckling surgery despite persistent CBD. These results are similar to those obtained using UBM by Lávaque et al. [10], Xu W-W et al. [11], and Murta Fet et al. [12]. Mandava et al. [13] utilized a sectorial and anterior buckle to abut the cyclodialysis cleft, 2 mm away from the limbus. After cleft closure, the scleral buckle was removed. Portney et al. used a sectorial and anterior buckle to abut the CBD with previous cryotherapy in the area where the buckle was going to be placed [14]. Inukai et al. also showed successful management of hypotonic maculopathy using 360° scleral buckling [15]. One reason for this might be that cerclage inhibits the posterior displacement of fluid from the suprachoroidal space, thereby decreasing flow through the uveoscleral pathway [10]. 

Differences between the traumatic clefts described by Lávaque et al. and PVR-induced CBD may affect the increase in IOP and may be more relevant in traumatic cleft cases. This is probably due to the relatively healthy condition of the detached ciliary body epithelium after trauma compared with that in PVR-induced CBD. The residual vitreous at the vitreous base provides a scaffold for membranes containing proliferating cells and a deposited extracellular matrix [16]. These membranes also incorporate the ciliary epithelium, which produces the aqueous humor, and prevent the reattachment of the ciliary body. 

In the literature, there is no correlation between the extension of the cleft and the severity of hypotony to suggest that hypofunction of the ciliary body epithelium plays a significant role in persistent hypotony [9,17,18]. 

Our results suggest that scleral buckling surgery might increase IOP in eyes with persistent severe hypotonia and CBD caused by PVR or trauma; in some cases, the increase in IOP might be sufficient to eliminate or prevent postsurgical hypotony. Our work was based on a small case series. Further studies are needed to analyze ciliary body conditions after PVR-induced retinal detachment to improve outcomes in eyes with severe and currently untreatable end-stage PVR-induced retinal detachment.

## Figures and Tables

**Table 1:** Patient data.

Patient No.	Sex Age	BCVA before the Primary Surgery (Snellen, logMAR)	Final BCVA, 12 Months Post-Op. (Snellen, logMAR)	Primary Pathology and Previous Surgical Procedures	IOP Baseline	IOP Final, 12 Months Post-Op.	CB Baseline	CB Final, 12 Months Post-Op.
**1**	**M** **67**	HM (2.3 logMAR)	0.01 (2.0 logMAR)	**Globe injury with traumatic retinal detachment**: 1. phacoppV+silicone oil 2. reppV+silicone oil exchange 3. 360° scleral buckling	6	8	CB detached in 3 quadrants	CB detached in 3 quadrants
**2**	**F** **65**	0.05 (1.30 logMAR)	0.05 (1.30 logMAR)	**Retinal detachment with subretinal hemorrhage, VH**: 1. plombage 2. phacoppV+gas 3. ppV+silicone oil 4. 360° scleral buckling	4	8	CB detached in 4 quadrants	CB detached in 4 quadrants
**3**	**F** **48**	HM (2.3 logMAR)	0.03 (1.52 logMAR)	**Retinal detachment**: 1. phacoppV (BIL)+gas 2. 360° scleral buckling 3. ppV+silicone oil + IOL reposition 4.ppV+silicone oil removal	6	14	CB detached in 1 quadrant	CB detahced in 1 quadrant
**4**	**F** **66**	0.1 (1.0 logMAR)	0.25 (0.60 logMAR)	**Retinal detachment**: 1. phacoppV+silicone oil 2. ppV+silicone oil removal 3. ppV+silicone oil 4.360° scleral buckling +silicone oil removal	5	14	CB detached in 3 quadrants	CB detached in 3 quadrants
**5**	**F** **82**	HM (2.3 logMAR)	HM (2.3 logMAR)	**Retinal detachment:** 1.ppV+gas 2.ppV+silicone oil 3.ppV+silicone oil removal 4.ppV+silicone oil 5.360° scleral buckling	4	6	CB detached in 4 quadrants	CB detached in 4 quadrants
**6**	**F** **66**	HM (2.3 logMAR)	0.05 (1.30 logMAR)	**Retinal detachment** 1.ppV+gas 2.360° scleral buckling	4	11	CB detached in 4 quadrants	CB detached in 4 quadrants

No.—number, F—female, M—male, BCVA—best-corrected visual acuity, BIL—Bag in the Lens, phacoppV—phacovitrectomy, CB—ciliary body, HM—hand motion, plombage—the surgical protocol; drainage, filtered air injection to restore the tonus of the eye, followed by cryotherapy of single horseshoe tear and placing of primary radial sponge 3 mm-wide (FCI S.A.S., France) in inferior-temporal quadrant using Ethibond 5/0 mattress suture. VH—vitreous hemorrhage.

## Data Availability

The data presented in this study are available within this article.
